# Semi-subterranean environment and soil metagenomic datasets of the Gyeongju Seokbinggo (stone ice storage) in South Korea

**DOI:** 10.1016/j.dib.2022.108308

**Published:** 2022-05-21

**Authors:** YoungHee Kim, Boa Lim, JiHee Park, SooJi Kim

**Affiliations:** Restoration Technology Division, National Research Institute of Cultural Heritage, 132 Munji-ro, Yuseong-gu, Daejeon 34122, Republic of Korea

**Keywords:** Semi-subterranean environment, Stone architecture, Metagenome analysis

## Abstract

The Seokbinggo is an ice cellar made of stone to store ice in the 1700s. The Seokbinggo, a traditional Korean stone architecture, can keep ice collected in winter until summer because the semi-subterranean structure utilizes the natural environment, and the insulation design is effective. However, these structures and scientific designs are not used as ice storage and are easily damaged by biological contamination. We present the environmental data of the inside and the metagenomic dataset of the soil samples from the Seokbinggo. Next-generation sequencing was carried out on an Illumina MiSeq platform. The raw sequence data used for analysis is available in NCBI under the Sequence Read Archive (SRA) with BioProject No. PRJNA727939 and SRA accession Nos. SRX10976613, SRX10976614, SRX10976615, SRX10976616, SRX10976617, SRX10976618, SRX10976619, SRX10976620. Environmental data, including data from Korea Meteorological Administration, is available in the Mendeley data repository with DOI: 10.17632/2r8gpg7pxn.1.

## Specifications Table

**Table:** 

Subject	Environmental science, Microbiology
Specific subject area	This data provides information on the semi-subterranean environment and soil microorganisms inside the Seokbinggo, Gyeongju city in South Korea.
Type of data	Table Figure
How data were acquired	Real-time temperature and relative humidity were measured by installing testo 174 H and 175 H1 data loggers at five points inside the Seokbinggo. This data acquisition period is from October 18, 2019, to October 21, 2020. Soil samples were collected from the four corners of Seokbinggo for use in NGS analysis. Amplicons for the 16S region and ITS region were sequenced using the Illumina MiSeq platform (300 × 2 pair-end).
Data format	Raw (environment data and sequencing data) Analysed
Parameters for data collection	Parameters for the environmental data contain temperature and relative humidity. The metagenomic data of bacteria and Eukaryota were prepared by amplifying the V3-V4 region of the 16S rRNA gene and the ITS region sequenced on the Illumina MiSeq platform.
Description of data collection	Environment data were collected from October 18, 2019, to October 21, 2020, from five points inside the Seokbinggo. These data were used to calculate average, maximum, and minimum values. Metagenomic DNA was extracted from the Seokbinggo's soil samples. Next-Generation Sequencing on the Illumina MiSeq platform was done and analysis was carried out using de novo genome assembly.
Data source location	Institution: National research institute of cultural heritage City: Gyeongju, Gyeongsangbuk-do Country: the Republic of Korea Environmental data were collected from October 18, 2019, to October 21, 2020, from five points inside the Seokbinggo, and soil samples were obtained from four sites inside the Seokbinggo. Latitude and longitude coordinates for collected samples: 35°49′59.38″ N 129°13′26.29″ E
Data accessibility	Repository name: Mendeley Data and NCBI SRA. The environment data is available in Mendeley with the DOI: 10.17632/2r8gpg7pxn.1 and the sequencing data is available in NCBI under the Sequencing Read Archive (SRA) with the BioProject No. PRJNA727939 and SRA accession Nos. SRX10976613, SRX10976614, SRX10976615, SRX10976616, SRX10976617, SRX10976618, SRX10976619, SRX10976620. Direct URL to data: https://data.mendeley.com/datasets/2r8gpg7pxn/1 https://www.ncbi.nlm.nih.gov/bioproject/PRJNA727939 https://www.ncbi.nlm.nih.gov/sra/SRX10976613[accn] https://www.ncbi.nlm.nih.gov/sra/SRX10976614[accn] https://www.ncbi.nlm.nih.gov/sra/SRX10976615[accn] https://www.ncbi.nlm.nih.gov/sra/SRX10976616[accn] https://www.ncbi.nlm.nih.gov/sra/SRX10976617[accn] https://www.ncbi.nlm.nih.gov/sra/SRX10976618[accn] https://www.ncbi.nlm.nih.gov/sra/SRX10976619[accn] https://www.ncbi.nlm.nih.gov/sra/SRX10976620[accn]

## Value of the Data

• This dataset is the only building in the Wolseong site that maintains its original shape and can be used as important data for site excavation.
• Since it maintains its original shape among Seokbinggo in South Korea, it can be used as data for conservation research and comparison with other local Seokbinggos.
• This dataset represents the taxonomic profile of microbial communities and environmental data within the semi-subterranean architecture.
• This data can be used as useful basic data for researchers who study the correlation between environmental factors and microbial growth.
• It can be used as comparative research data for microbial diversity studies inside caves or tombs.
• This data can also be used to study the secondary metabolites of microorganisms and their side effects.

## 1 Data Description

**Fig. 1:**
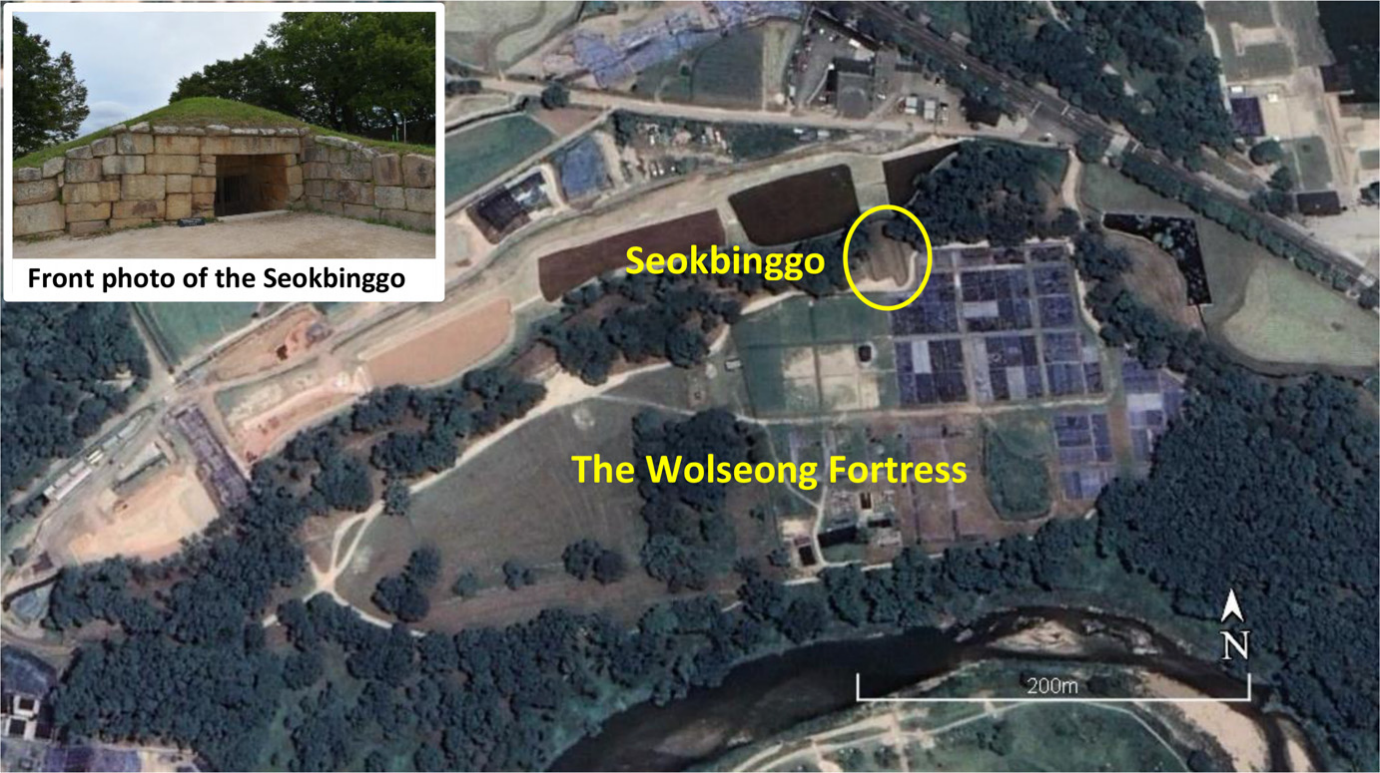
Location of Seokbinggo at the Wolseong Fortress site in Gyeongju city [3].

**Fig. 2:**
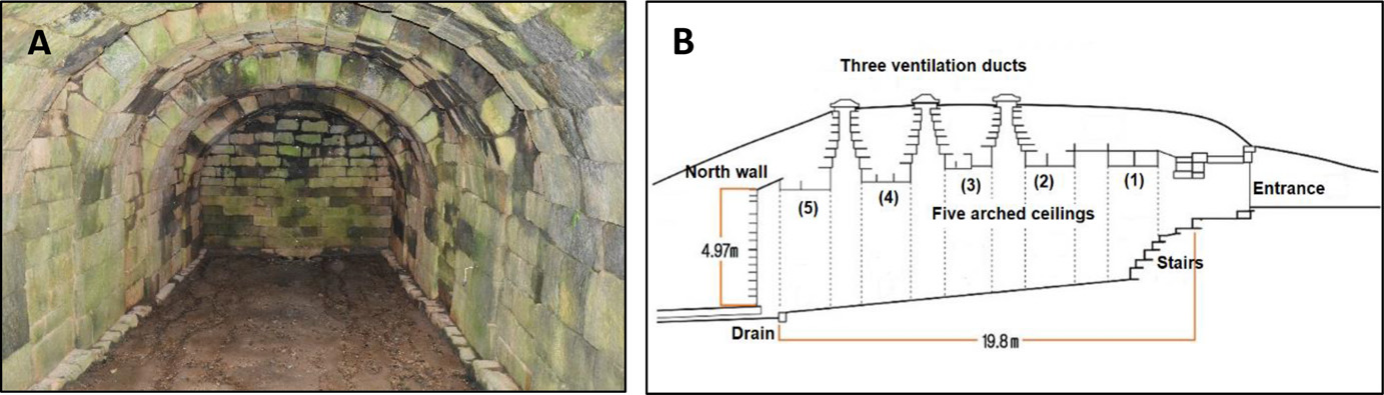
Seokbinggo interior photo (A) and the cross-sectional drawing (B).

Located on the northern hill of Wolseong Fortress in Gyeongju city, this structure was used to store ice during the Joseon Dynasty (Fig. 1). The storage facility can be entered by a descending stairway on its southern side and has three ventilation ducts installed on its arched roof (Fig. 2. B). This architecture was called the Seokbinggo. The existing location of Seokbinggo is built long from north to south on a sloping topography high in the south and low in the north, and when viewed from a distance, it looks like a long tomb (Fig. 1). Its walls and arched ceiling of the interior are made of granite. The floor is slanted, which allowed melted ice to funnel out. At the end of the sloped floor is a drain. The floor slopes facilitate the flow of water from melting ice. The ice room is rectangular, with a length of 19.86 m, a width of 5.94 m, and an area of 117.9 m^2^. The height from the floor to the arched ceiling is 4.97 m (Fig. 2. B). The Seokbinggo of this size is the largest among the remaining ice storages [1,2]. Fig. 2 shows the internal state and the structure of Seokbinggo.

We provide the real-time datasets collected from October 18, 2019, to October 21, 2020, from five points inside the Seokbinggo. These data parameters include air temperature and relative humidity. All parameters were measured at five points inside Seokbinggo using Testo data loggers with temperature and relative humidity sensors. A total of 84,693 measurements of each parameter were recorded from October 18, 2019, to October 21, 2020. 8870 data per parameter were collected at one site inside Seokbinggo, but data of location M3-L were collected only 4863. This is because moisture seeped into the vent at the top of the Seokbinggo and the measurement device located at the low position was damaged. This dataset contains temperature and relative humidity data obtained at each point inside the Seokbinggo, as well as raw data from the Gyeongju observatory of the Korea Meteorological Administration [4]. This dataset is available in Mendeley Data Repository (DOI: 10.17632/2r8gpg7pxn.1). These data were used to calculate average, maximum, and minimum values (Table 1). The Seokbinggo, a traditional Korean stone heritage, can keep ice collected in winter until summer because the subterranean structure utilizes the natural environment, and the insulation design is effective. However, these structures and scientific designs are not used as ice storage and are easily damaged by biological contamination.

**Table 1:** Average temperature and humidity from October 18, 2019, to October 21, 2020, of the Seokbinggo and Gyeongju observatory.

			Device installation location inside Seokbinggo					Gyeongju
Environment factor			F1	M2	M3	M4	B5	KMA
Temperature (°C)	L	Mean	12.8	12.6	–	12.9	12.4	13.8
		Max	23.9	24.1	–	24.8	23.7	35.6
		Min	2.7	2.6	–	2.7	2.7	−7.4
	M	Mean	13.4	13.1	13.5	12.9	12.7	
		Max	25.0	24.6	25.2	24.2	24.1	
		Min	3.4	3.3	3.0	3.1	2.7	

Relative humidity (%)	L	Mean	93.4	93.3	–	94.9	95.0	69.7
		Max	99.9	99.9	–	99.9	99.9	99.0
		Min	46.1	48.8	–	64.5	55.8	10.8
	M	Mean	92.5	93.1	93.5	93.3	94.5	
		Max	99.9	99.9	99.9	99.9	99.9	
		Min	35.4	43.5	46.6	45.8	44.1	

**Fig. 3:**
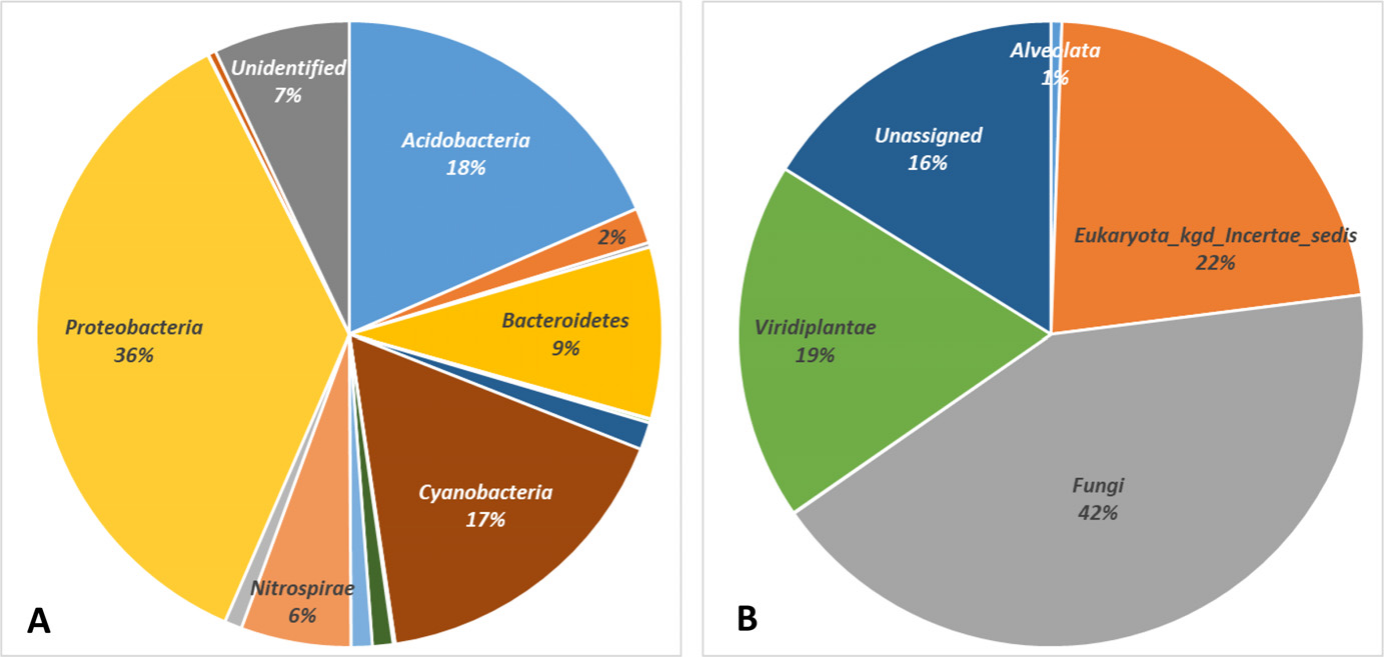
Charts of the taxonomic composition about 16S rRNA (A) and ITS region (B).

This article contains data on microbial communities formed in the soil of four points within the Seokbinggo. The first two soil samples (KJ.SBG.1 and 4) are located in the southern part of the inner Seokbinggo. Another two soil samples (KJ.SBG.2 and 3) are located in the northern part, about 20 m from the entrance of the inner Seokbinggo. Raw sequencing data of the sample KJ.SBG.1 contain 11,501 and 81,577 reads for 16S region and ITS region. Other samples of KJ.SBG.2, and KJ.SBG.3, and KJ.SBG.4 were sequenced for the 16S and ITS region, and Table 2 shows the total number of sequencing reads and details about the metagenome analysis for each sample. Also, Table 2 provides for the corresponding operational taxonomical unit (OTU) values and the Shannon diversity index for all taxonomical levels from kingdom to species. Data were presented as taxonomic and functional profiles, as shown in Tables 3 and 4, respectively. The relative abundance of bacteria and Eukaryota was depicted at the phylum levels (Fig. 3). The raw FASTQ files generated in this study were deposited at the NCBI SRA database under BioProject PRJNA727939.

**Table 2:** Details about the metagenome analysis for soil samples in Seokbinggo. the estimated richness and diversity indices at 3% dissimilarity of a threshold.

Sample name		No. of Sequences	OTUs	Chao1	Shannon Index	Inverse Simpson	Good's Coverage
16S	KJ.SBG.1	11,501	662	716.5	7.29316	0.98290	0.99044
	KJ.SBG.2	12,423	571	653.0	6.65520	0.97093	0.99018
	KJ.SBG.3	17,262	628	715.8	6.49395	0.95121	0.99398
	KJ.SBG.4	17,539	700	752.0	7.11659	0.98062	0.99396

ITS	KJ.SBG.1	81,577	356	359.0	6.03163	0.96091	0.99995
	KJ.SBG.2	53,084	213	213.0	5.91756	0.96023	0.99998
	KJ.SBG.3	41,871	124	124.0	5.13385	0.91496	0.99998
	KJ.SBG.4	52,977	254	257.3	5.49728	0.93305	0.99991

**Table 3:** The taxonomic composition of 16S rRNA region.

Kingdom	Phylum	KJ.SBG.1	KJ.SBG.2	KJ.SBG.3	KJ.SBG.4
Archaea	Euryarchaeota	2	6	17	5
	Thaumarchaeota	1	1	0	1

Bacteria	Acidobacteria	2349	2723	2463	3300
	Actinobacteria	141	377	287	270
	Armatimonadetes	45	23	1	70
	Bacteroidetes	822	397	1993	1979
	Candidatus Melainabacteria	3	5	5	4
	Chlamydiae	18	2	50	38
	Chloroflexi	176	107	319	226
	Cyanobacteria	1642	2022	3930	2198
	Deinococcus-Thermus	10	10	4	0
	Dictyoglomi	0	1	3	5
	Firmicutes	171	124	185	144
	Gemmatimonadetes	135	87	240	180
	Nitrospirae	561	864	836	1081
	Planctomycetes	189	99	100	139
	Proteobacteria	4232	4889	5527	6512
	Spirochaetes	0	5	0	4
	Thermodesulfobacteria	1	1	7	3
	Verrucomicrobia	55	66	67	41

Unassigned	Other	948	614	1228	1339

Total		11,501	12,423	17,262	17,539

**Table 4:** The taxonomic composition of ITS region.

Kingdom	Phylum	KJ.SBG.1	KJ.SBG.2	KJ.SBG.3	KJ.SBG.4
Alveolata	Ciliophora	551	735	0	0

Eukaryota_kgd_Incertae_sedis	Unidentified	20,460	9312	8159	13,548

Fungi	Other	65	0	62	27
	Aphelidiomycota	41	0	0	15
	Ascomycota	23,956	14,260	12,299	20,499
	Basidiomycota	2508	2198	1869	560
	Chytridiomycota	338	306	1299	51
	Monoblepharomycota	0	538	0	0
	Mortierellomycota	2665	707	927	283
	Mucoromycota	0	0	0	14
	Rozellomycota	182	0	0	98
	Unidentified	3134	4067	1813	2374

Protista	Unidentified	17	0	0	63

Rhizaria	Cercozoa	3	0	0	4

Viridiplantae	Other	0	1	3	0
	Anthophyta	6811	8155	11,097	6
	Bryophyta	10	0	0	0
	Chlorophyta	7113	5092	2360	1776
	Streptophyta	11	7	0	0

Unassigned	Other	13,712	7703	1983	13,659

Total		81,577	53,084	41,871	52,977

## 2 Experimental Design, Materials and Methods

### 2.1 Survey Area

The Seokbinggo is located within the Wolseong Fortress in the Gyeongju Historic Areas. Gyeongju Historic Area was designated as a World Heritage Site in 2000. Gyeongju is a major city that embodies the history and culture of Silla (57 B.C. – A.D. 935), the golden age of ancient Korean civilization. Gyeongju's historic area has the famous palace site, Wolseong Fortress (Moon Fortress). Encircling the palace compounds, walls were built of stone and mud to the north, east, and west, while natural cliffs and a flowing stream formed the southern border. Stone terraces and moats were built along the northern and western borders, and a gate on the eastern border led to the Palace of the Crown Prince [2]. The study area is located on the northern border. The palace and other buildings do not remain, and excavations are underway at the palace site.

### 2.2 Data Acquisition and Sample Collection

**Fig. 4:**
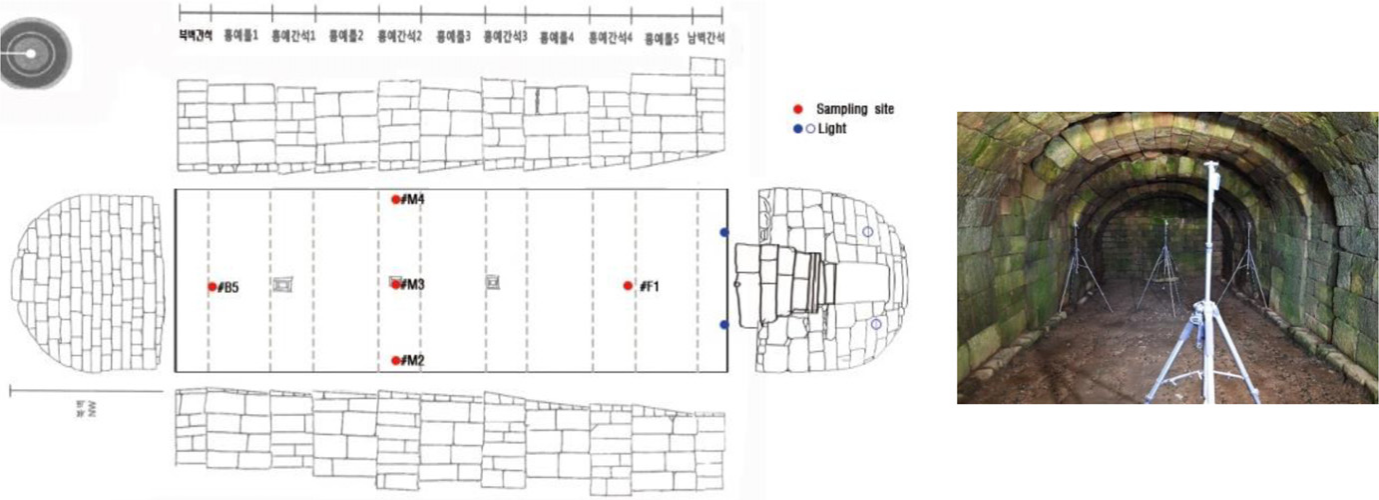
The installation location of environmental survey equipment inside the Seokbinggo.

This data acquisition period is from October 18, 2019, to October 21, 2020. We also applied the same period of time for the public database (data.kma.go.kr) [4]. TESTO Temperature/Relative humidity data loggers (testo 174 H and testo 175 H1, Germany) were installed on the front, middle, and rear sides in the Seokbinggo, respectively, (Fig. 4). The exact location of the equipment is shown in Table 5. The instrumental characteristics of data logger, testo 174 H, and testo 175 H1 are given in Table 6.

**Table 5:** The installation location of environmental survey equipment inside the Seokbinggo.

	Distance (m)		Height (m)		
Site	North wall	West wall	Position	From floor	Equipment
F1	14.350	2.935	L	1.290	testo 175 H1
			M	2.697	testo 175 H1

M2	7.054	0.536	L	1.384	testo 174 H
			M	2.870	testo 175 H1

M3	6.831	2.935	L	1.245	testo 174 H
			M	2.619	testo 175 H1

M4	6.980	5.289	L	1.331	testo 174 H
			M	2.697	testo 175 H1

B5	1.662	2.935	L	1.183	testo 174 H
			M	2.592	testo 175 H1

**Table 6:** Instrumental characteristic of the testo 174 H and testo 175 H1 data loggers.

Specification	testo 174 H	testo 175 H1
Parameters	Temperature/Humidity	Temperature/ Humidity/ Dew Point
Probe type	NTC/ Humidity sensor	NTC/ Humidity sensor
Number of channels	2	2
Measurement range	−20 to 70 °C / 0–100%	−20 to 55 °C / 0–100% / −40 to 50 °C
Accuracy	±0.5 °C / ± 3.0% (at 25 °C)	± 0.4 °C / ± 2.0% (at 25 °C)
Resolution	0.1 °C / 0.1%	0.1 °C / 0.1%
Memory	16,000 readings	1,000,000 readings

**Fig. 5:**
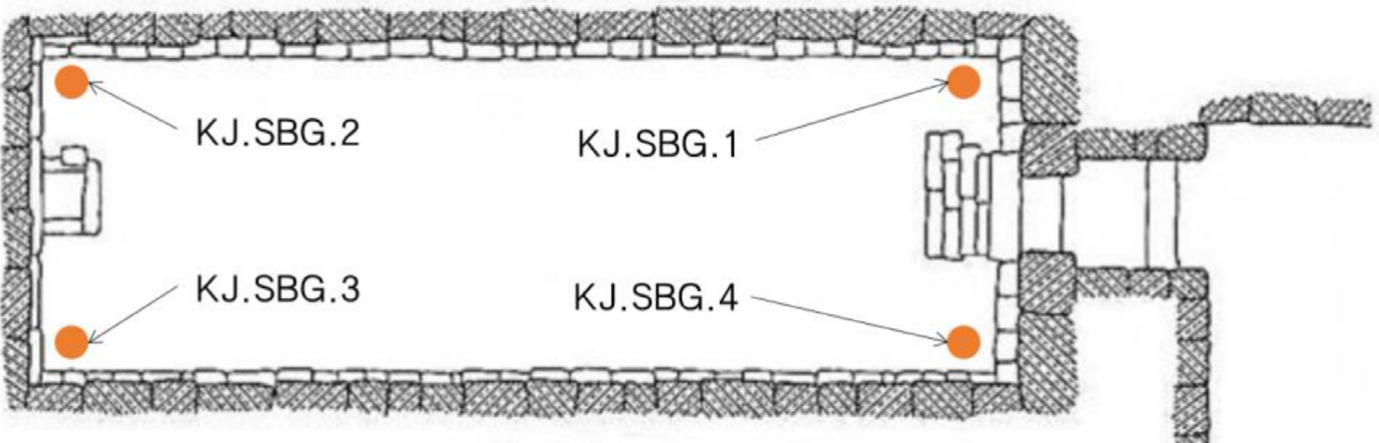
Soil sampling sites for NGS analysis inside the Seokbinggo.

Soil samples were collected from the four corners of Seokbinggo for use in NGS analysis (Fig. 5). At each point, soil samples were collected from the depth of 10 cm in 50 mL containers. The bottom was lowered inward from the inlet that samples KJ.SBG.2 and KJ.SBG.3 farther from the inlet had a higher moisture content than the other samples KJ.SBG.1 and KJ.SBG.4.

### 2.3 Data Processing

**Fig. 6:**
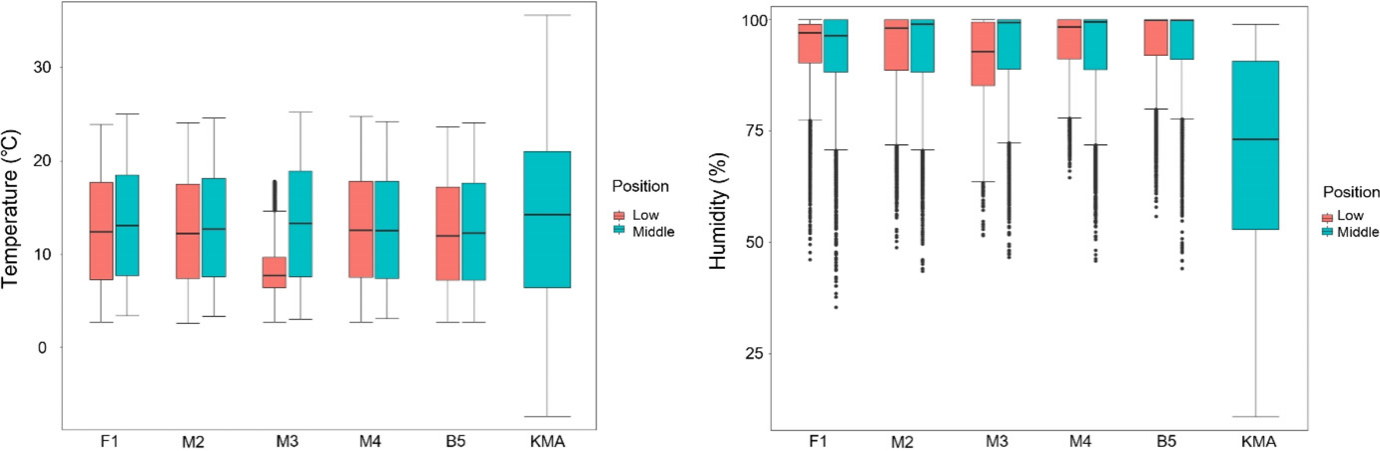
Boxplot chart of the temperature and humidity according to the measurement location (M3-L: data loss).

The Excel 2016 program was used to convert the collected data into average, maximum, and minimum values. We calculated the average, maximum, and minimum values using the pivot table function of the Excel program and confirmed the temperature and relative humidity between the inside of Seokbinggo and the public database. The M3-L data were not used in the calculation due to missing data. Using the RStudio 1.2.1335 program, the temperature and humidity change according to the measurement location was shown in the boxplot chart (Fig. 6).

Gene amplification and sequencing were performed by Macrogen Ltd. (Seoul, Korea), and the final results were sequenced using the Illumina MiSeq platform (Illumina Inc., San Diego, CA, USA) [5]. Forward primer 341F and reverse primer 805R for 16S rRNA amplification were used to amplify the V3-V4 region of the bacterial 16S rRNA. ITS rRNA was amplified using forward primer 3F and reverse primer 4R. Operational taxonomic unit (OTU) clustering was used in the de novo (CD-HIT) OTU picking method. Sequences were assigned to taxonomy using a classifier trained on NCBI 16S rRNA BLAST database and ITS rRNA UNITE database version 8.2 with the read matching to the Refs. [6,7].

## Ethics Statement

This data is NOT relevant for human projects and animal studies.

## CRediT authorship contribution statement

**YoungHee Kim:** Conceptualization, Investigation, Supervision, Writing – original draft. **Boa Lim:** Data curation, Investigation. **JiHee Park:** Project administration, Investigation. **SooJi Kim:** Validation, Writing – review & editing.

## Declaration of Competing Interest

The authors declare that they have no known competing financial interests or personal relationships that could have appeared to influence the work reported in this paper.

## Data Availability

Semi-subterranean environment data of the Gyeongju Seokbinggo in South Korea (Original data) (Mendeley Data) Gyeongju Seokbinggo's soil samples Metagenome (Original data) (NCBI SRA).
